# Histopathological changes of lymphatic malformation after bleomycin injection

**DOI:** 10.5935/0004-2749.20230048

**Published:** 2023

**Authors:** Yasser H Al-Faky, Hind Manaa Alkatan

**Affiliations:** 1 Department of Ophthalmology, College of Medicine, King Saud University, Riyadh, Saudi Arabia.; 2 Department of Pathology, College of Medicine, King Saud University, Riyadh, Saudi Arabia.

**Keywords:** Lymphangioma, Bleomycin, Lymphatic abnormality, Linfangioma, Bleomicina, Anormalidade linfática

## Abstract

Lymphatic malformation is a rare orbital tumor that used to be treated surgically, with high complication rates, or recently with intralesional bleomycin injection. We report for the first time the histopathological changes of eyelid lymphatic malformation after water-soluble intralesional bleomycin injection in a 20-year-old woman who had unsuccessful orbital surgical debulking during childhood. The changes confirmed the assumption of fibrosis induced by intralesional bleomycin injection. The minimal bleeding during surgical intervention made it much easier than the usual lymphatic malformation bloody procedure, without postoperative recurrences and with favorable aesthetic outcomes.

## Abstract (pt)

A malformação linfática é um tumor orbital raro que costumava ser tratado cirurgicamente, com alta taxa de complicações. Mais recentemente, passou a ser tratado com uma injeção intralesional de bleomicina. Este é o primeiro relato sobre as alterações histopatológucas da malformação linfática palpebral após uma injeção intralesional de bleomicina hidrossolúvel em uma mulher de 20 anos de idade que sofreu uma cirurgia malsucedida de *debulking* orbital durante a infância, confirmando a suposição de fibrose induzida por injeções intralesionais de bleomicina. O sangramento mínimo durante a intervenção cirúrgica tornou esta muito mais fácil que o procedimento sangrento habitual, sem recidivas pós-operatórias e com desfechos estéticos favoráveis.

## INTRODUCTION

Lymphatic malformation (LM) is a benign lymphatic vascular anomaly that rarely affects the orbit^(1)^. As surgical management carries high complication rates, intralesional bleomycin injection (ILBI) has been adopted as a primary treatment modality for orbital LM^(2,3)^. Although the fibrotic changes induced by bleomycin had been confirmed by a histopathological study in 1987, it was a fat emulsion^(4)^. The effect of water-soluble bleomycin in orbital LM is attributed to tissue sclerosing, which had not been confirmed yet by a histopathological study^(3)^. We present, for the first time, the efficient surgical debulking of a case of orbital LM with eyelid extension after ILBI, appended with the histopathological and immunohistochemistry confirmation of the sclerosing effect of water-soluble ILBI.

## CASE REPORT

**Figure 1:**
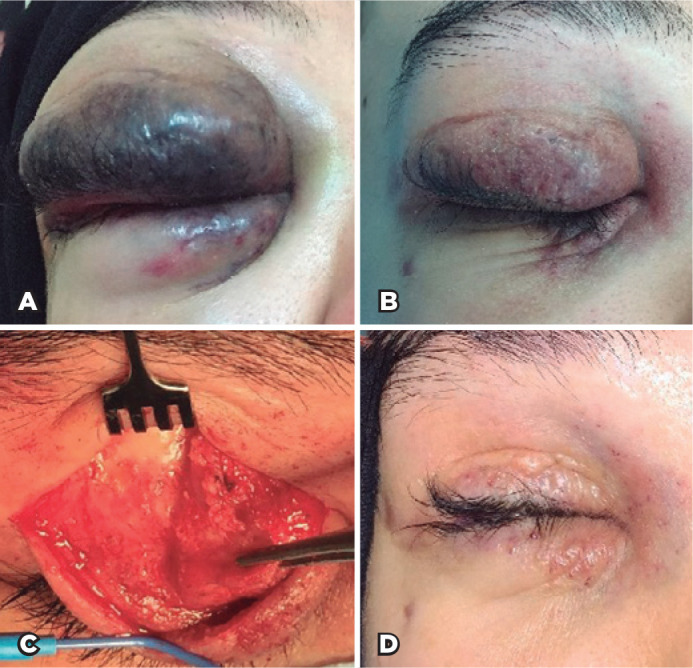
(A) External photo of extensive LM of the eyelids at presentation. (B) External photo of the eyelids after 5 sessions of ILBI shows partial regression of LM. (C) Intraoperative photos showing the residual fibrotic lesion during excision. Note the minimal bleeding during the procedure. (D) Postoperative photo showing improvement of the thickness and color of the eyelid.

A 20-year-old woman was referred to a tertiary hospital as a case of right orbital LM with eyelid and subconjunctival extension since early childhood. She had unsuccessful orbital surgical debulking twice at the age of 4 and 5 years. Histopathological report confirmed orbital LM. She presented with extensive disfigurement of the right periorbital area. On examination, she had extensive dark bluish soft lesions involving the whole right upper eyelid, with color fading toward the eyebrow and complete ptosis. The lower eyelid showed a less extensive lesion involving the medial aspect and extending to the medial canthus area (Figure 1A). Extraocular muscle motility showed limitation in all positions of gaze with 20/100 visual acuity. B-scan ultrasonography revealed a predominantly microcystic eyelid and orbital lesions.

The patient received 5 consecutive ILBIs every 6-8 weeks at variable doses ranging from 3 to 5 mg per injection, which did not exceed 0.5 mg/kg of body weight. The injections were given in the eyelids, orbit, and cheek. She reported post-injection pain and increase in the size of the lesion after the first and second injections. No systemic complications were reported. The upper eyelid lesion showed partial response, and the patient was concerned of disfigurement (Figure 1B). Hence, surgical debulking of the upper eyelid lesion was performed 16 months after the first injection. The surgical debulking was performed with ease in spite of the unclear surgical planes because of minimal bleeding. A pale-colored LM tissue with gritty sensation was noticed during surgical dissection (Figure 1C). The patient had a smooth postoperative course with stable results >3 years of follow-up and satisfactory eyelid cosmetic outcome (Figure 1D).

**Figure 2:**
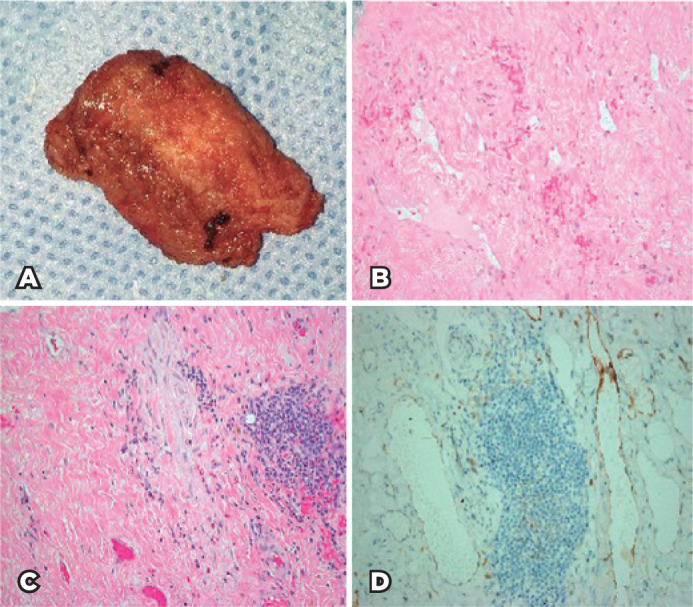
(A) Gross photo showing the excised lesion with fibrotic consistency. (B) Histopathological photo of the residual orbital LM with extensive fibrosis and residual narrow small endothelial-lined vascular spaces (original magnification ´200; hematoxylin and eosin staining). (C) Histopathological image of residual partially occluded orbital LM with focal aggregate of lymphocytes typically seen adjacent to LM spaces (original magnification ´200; hematoxylin and eosin staining). (D) The lymphatic nature of the residual endothelial-lined spaces confirmed by IHC staining as outlined in this image (original magnification ´200, podoplanin).

The excised tissue (Figure 2A) was sent for histopathological evaluation, which revealed residual orbital lymphangioma with focal dense lymphocytic infiltrate adjacent to the lymphangiomatous spaces, occluded blood vessels, and fibrosis. Most of the histological sections showed extensive fibrosis and only few residual narrow vascular spaces lined by endothelial cells highlighted by the expression of staining to the immunohistochemical (IHC) marker for the lymphatic endothelium (podoplanin; Figure 2B-D).

## DISCUSSION

Orbital LM is relatively rare representing, 0.3%-4% of all orbital tumors^(2)^. It may be located deeply within the orbit or combined with subcutaneous components^(1)^. Despite being benign, it draws attention because of the pressure effect on the adjacent structures induced by spontaneous or traumatic bleeding, the infiltrative nature with difficult surgical excision, or aesthetic reasons^(1)^. Owing to the high rate of complications after surgical treatment and risk of recurrences, ILBI sclerotherapy was introduced in 1977 as a treatment option for LM^(5)^. Recently, ILBI has become the first line management method for orbital LM after being advocated as a successful treatment for refractory orbital LM^(3)^.

The presented case underwent surgical debulking of orbital LM twice during early childhood, which were not successful in alleviating neither the mass effect nor the disfigurement caused mainly by the eyelid component. ILBI was performed in multiple sessions but achieved only partial response due to the predominance of the microcystic variant of the lesion. Hence, surgical debulking was offered to improve the cosmetic shape of the eyelid. Although surgical intervention in LM is usually a bloody procedure, the fibrotic effect of ILBI made the surgical debulking much easier with minimal bleeding. However, the surgical planes were not identifiable because of the infiltrative and unencapsulated nature of LM^(2)^. The lack of macrocystic variant and bleeding did not suggest the use of negative pressure after surgery, which was suggested to collapse the lymphatic cyst^(6)^.

The classic histopathological features of LM included numerous dilated lymphatic channels of varying sizes within a fibro-collagenous stroma with lymphoid cell aggregates resembling lymphoid follicles. CD34, which is a vascular endothelial marker has been used to highlight the vascular and lymphatic channels^(4)^. However, more specific lymphatic endothelial markers have been advocated to better identify orbital vascular lesions and to confirm the lymphatic nature of the vascular spaces in LM, such as podoplanin, D2-40, and vascular endothelial growth factor receptor 3^(7,8)^. In our case, the lymphatic nature of the residual spaces was confirmed using podoplanin, outlining the residual narrow lymphatic channels with lymphocytic infiltrate adjacent to them. The occluded channels and extensive fibrosis confirmed the presumed fibrotic effect of ILBI in our orbital LM. The natural history of long-standing lymphangioma might show mild fibrotic changes histopathologically. However, in this case, the fibrosis was marked (with thick and wavy collagen fibers), widely spread across the whole excised tissue and resulting in narrowed and occluded endothelial-lined spaces and vascular channels, as shown in our figures, which support ILBI-induced fibrosis.

Bleomycin is classified as an “antitumor antibiotic” made by the bacterium *Streptomyces verticillus*, and the cytotoxic effect is attributed to the prevention of DNA synthesis by inhibiting incorporation of thymidine into DNA strands resulting in strand breaks^(9)^. In LM, the exact role of bleomycin in inducing endothelial damage and fibrosis is poorly understood; however, the sclerosing effect is more evident with bleomycin emulsion than with bleomycin aqueous solution^(10,11)^. To our knowledge, a single case of LM histopathology evaluation after ILB injection was published in English literature in 1987, but the bleomycin used was in the form of an emulsion, and the lesion was in the neck^(4)^. In our report, the lesion was in the eyelid as an extension of orbital LM, and the bleomycin used was in the aquous form, which is the form commonly used nowadays. ILBI induces fibrosis of orbital LM and facilitates further surgical debulking, if needed.

In conclusion, ILBI is an effective treatment for inducing fibrosis and facilitating surgical debulking of orbital LM.
